# Ultra–sensitive droplet digital PCR for detecting a low–prevalence somatic *GNAQ* mutation in Sturge–Weber syndrome

**DOI:** 10.1038/srep22985

**Published:** 2016-03-09

**Authors:** Yuri Uchiyama, Mitsuko Nakashima, Satoshi Watanabe, Masakazu Miyajima, Masataka Taguri, Satoko Miyatake, Noriko Miyake, Hirotomo Saitsu, Hiroyuki Mishima, Akira Kinoshita, Hajime Arai, Ko–ichiro Yoshiura, Naomichi Matsumoto

**Affiliations:** 1 Department of Human Genetics, Yokohama City University Graduate School of Medicine, Yokohama, Japan; 2 Department of Medical and Clinical Science, Gunma University Graduate School of Medicine, Gunma, Japan; 3 Department of Human Genetics, Nagasaki University Graduate School of Biomedical Sciences, Sakamoto, Nagasaki, Japan; 4 Department of Neurosurgery, Juntendo University Graduate School of Medicine, Tokyo, Japan; 5 Department of Biostatistics, Graduate School of Medicine, Yokohama City University, Yokohama, Japan

## Abstract

Droplet digital PCR (ddPCR), a method for measuring target nucleic acid sequence quantity, is useful for determining somatic mutation rates using TaqMan probes. In this study, the detection limit of copy numbers of test DNA by ddPCR was determined based on Poisson distribution. Peptide nucleic acid (PNA), which strongly hybridises to target lesions, can inhibit target amplification by PCR. Therefore, by combination of PCR with PNA and ddPCR (PNA–ddPCR), the detection limit could be lowered. We reanalysed a somatic *GNAQ* mutation (c.548G > A) in patients with Sturge–Weber syndrome (SWS) using ddPCR and PNA–ddPCR. Importantly, among three patients previously found to be mutation negative by next–generation sequencing, two patients had the *GNAQ* mutation with a mutant allele frequency of less than 1%. Furthermore, we were able to find the same mutation in blood leukocyte or saliva DNA derived from four out of 40 SWS patients. Vascular anomalies and blood leukocytes originate from endothelial cells and haemangioblasts, respectively, which are both of mesodermal origin. Therefore, blood leukocytes may harbour the *GNAQ* mutation, depending on the time when the somatic mutation is acquired. These data suggest the possibility of diagnosis using blood DNA in some patients with SWS.

Sturge–Weber syndrome (SWS, MIM#185300) is a neurocutaneous disorder characterised by the following manifestations: 1) cutaneous vascular malformations (port–wine stains), 2) ocular vascular malformations leading to choroidal vascular abnormalities, glaucoma, buphthalmia and hemianopia, and 3) intracranial vascular malformation resulting in neurological impairment including seizures and intellectual disability^1^,^2^,^3^,^4^,^5^. The prevalence is estimated at approximately 1/20,000–1/50,000^1^. It has been suggested that SWS is likely to be caused by somatic mutations because its occurrence is sporadic with no heritability^6^. Recently, a somatic c.548G > A mutation in *GNAQ* [encoding guanine nucleotide–binding protein, Q polypeptide (MIM600998)] was indeed identified in 88 and 92% of patients with SWS and those with non–syndromic port–wine stains, respectively^3^. We also confirmed the presence of low–prevalence somatic *GNAQ* mutations in 12 of 15 SWS samples using deep sequencing (80%), and no other possible somatic *GNAQ* mutations were found^7^. In these two reports, mutant allele frequencies in brain samples ranged from 1.0 to 11.15%^7^. Both studies adopted the 1% cut–off line to detect mutant alleles in total sequence reads to excluding possible errors of PCR and read misalignment/mapping. Therefore, there is a possibility that extreme low–prevalence (<1%) mutations could be overlooked.

Droplet digital PCR (ddPCR) is a sensitive method enabling the accurate quantification of a target nucleic acid sequence^8^,^9^. In this method, individual DNA molecules from a sample are captured within water–in–oil droplet partitions^9^. Droplets containing mutant or wild–type allele(s) are discriminated using two color–fluorescent TaqMan probes and the numbers of target DNA copies are counted at the end point of PCR^8^,^10^. Poisson distribution is used to assay DNA molecule concentration using numbers of accepted total amplified and un–amplified droplets^9^. Peptide nucleic acid (PNA) is a DNA/RNA mimic that can be hybridised to target sequences and prevent PCR amplification of target regions^11^,^12^. By combination of PCR with PNA and ddPCR (PNA–ddPCR), it may be possible to successfully detect low–prevalent mutant alleles more sensitively than with ddPCR alone as only mutant alleles are amplified.

We present an investigation of the detection limit of ddPCR and PNA–ddPCR using a target low–prevalence somatic *GNAQ* mutation (c.548G > A) in patients with SWS^7^ who were previously analysed only with next–generation sequencing (NGS)^3^,^7^.

## Results

### Detection limit of ddPCR

The detection limit of ddPCR was determined using serial dilutions of cloned mutant DNA (*GNAQ* c.548G > A) in non–mutant DNA at levels of 10, 5, 1, 0.5, 0.25 and 0.1%, the copy numbers of which were “300, 150, 30, 15 and 7.5” (in 3000), respectively (Table 1). However, mutant alleles at a frequency of <0.25% (7.5 copies) could not be consistently detected. Therefore, <0.25% is gray (under the detection limit) rather than completely negative. The mutation could be consistently detected at 10, 5, 1, 0.5 and 0.25% (Table 1). We evaluated the reliability of the detection limit using another statistical method based on the binominal distribution, supporting the above detection limit (see Supplementary Data and Table S1). This result also indicated that we were able to detect mutant DNA with confidence to 0.25% (see Supplementary Table S1). Therefore, the detection limit of ddPCR was defined as 0.25%. Fractional abundance (FA) (denoting the proportion of the mutant allele frequencies by QuantaSoft) of the 0.25% positive control actually indicated 0.26–0.42% (Table 1).

### The *GNAQ* somatic mutation in patients with SWS detected by ddPCR

The *GNAQ* mutation (c.548G > A) was quantified by ddPCR in DNA of paired brain and either leukocytes or saliva samples from 15 SWS patients previously analysed by NGS^3^,^7^ (Table 2). Comparable results were produced by ddPCR and NGS in most samples. Of 18 of these samples (three from brain, 14 from blood and one from saliva) in which NGS had detected mutant allele frequencies of less than 1%, 17 showed mutant allele frequencies of less than 0.25% of FA (considered to be under the detection limit) while the remaining sample (the saliva DNA), showed a mutant allele frequency of 0.61% of FA (SWS8; Table 2).

### Detection limit of PNA–ddPCR

**Figure 1:**
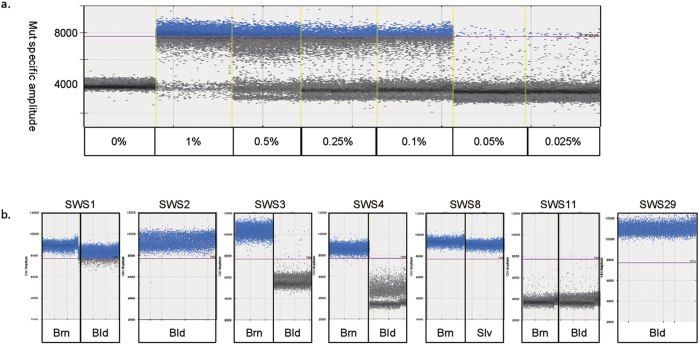
PNA–ddPCR analyses in SWS patients. Positive mutant–specific amplification was set to >1000 droplets with >7700 signal amplitude. (**a**) Detection limit of PNA–ddPCR. Mutant and wild–type clones were mixed serially in different ratios: 1, 0.5, 0.25, and 0.1% (mutant/mutant + wild–type). Ratios of 1–0.1% were positively amplified by PNA–ddPCR. (**b**) Mutation positivity was shown by PNA–ddPCR in 6 of 15 samples from SWS patients with <1% FA by ddPCR and in 1 of 25 samples from SWS patients for whom only blood samples were available in this study. Brn: Brain, Bld: Blood, Slv: saliva.

The sensitivity for PNA–ddPCR was determined using the same series of dilutions of mutant DNA. The mutation could be consistently detected at dilutions of 1, 0.5, 0.25 and 0.1% (Fig. 1a). However, 0.05–0.025% plasmid DNA showed inconsistently positive or negative results in each of triplicate experiments. The postulated reasons for this inconsistency for <0.1% plasmid DNA are as follows: 1) The mutant copy numbers at 0.1, 0.05 and 0.025% of 3000 copies would on average be 3, 1.5 and 0.75 copies. Therefore, the actual copy number of 0.025% can sometimes be one or zero (i.e. absent), depending on the aliquot used for PNA–ddPCR. 2) PCR contamination. PNA–ddPCR involves two rounds of PCR, so there is a risk of contamination. Based on the results of PNA–ddPCR using DNA from 30 healthy controls performed in triplicate, none of the results showed >1000 mutant droplets with >7700 signal intensity. Therefore, the criteria for positivity in detecting the mutant allele were set as >1000 mutant droplets with >7700 signal intensity. Considering the actual copy number of mutant DNA, 0.1% of loaded 1.5 × 10^−5^ ng clone DNA (equivalent to 3.0 × 10^3^ copies) is approximately three copies. Therefore, to obtain a positive result by PNA–ddPCR, more than three copies per reaction is required.

### *GNAQ* mutation detection by PNA–ddPCR in SWS patients

Four of the 21 samples from 15 SWS patients previously analysed by NGS were positive for the *GNAQ* mutation by PNA–ddPCR. We especially focused on brain samples (n = 3) and blood leukocyte or saliva samples (n = 14) that were under the detection limit (<0.25% of FA) by ddPCR, as well as one saliva sample with 0.61% FA. PNA–ddPCR newly revealed that two blood leukocyte samples (SWS1 and SWS2) and two brain samples (SWS3 and SWS4) were mutant-positive, as was saliva of SWS8 (Fig. 1b and Table 2). Blood leukocyte samples were analysed by PNA–ddPCR from additional 25 SWS patients whose affected tissues were unavailable for mutation screening. Of note, one blood sample was positive for the *GNAQ* mutation (SWS29; Fig. 1b and see Supplementary Table S2).

## Discussion

In this study, we have reached the conclusion that the detection limit of SNV by PNA–ddPCR was 3 copies in an aliquot. The data presented herein allow the determination of a hierarchy of sensitivity for novel mutation screening methods: 1% for NGS, 0.25% (7.5 copies) for ddPCR and 0.1% for PNA–ddPCR (3 copies). It thus follows that both 0.1% in 3.0 × 10^3^ copies and 0.01% in 3.0 × 10^4^ copies would produce a positive result because both scenarios would provide three copies in a single reaction. Importantly, therefore, if a greater overall quantity of DNA is used in PNA–ddPCR, the chance of detecting a low–prevalence mutation is increased. In practice, however, 3 × 10^4^ copies of DNA is the machine limit in one–well analysis using the Droplet Digital PCR XQ200 system. As such, there is the trade–off between DNA quantity and the detection limit of ddPCR. Stahl *et al*. showed that the detection limit of ddPCR for the analysis of chimerism was 0.01% (1.95 copies)^13^. They used two types of marker (different loci) to distinguish donor cells and recipient cells for this chimerism analysis^13^. Our target is a single-nucleotide variation and discrimination of the mutant from the wild type is rather difficult.

One advantage of NGS over ddPCR and PNA–ddPCR is that while the latter two techniques target known genomic changes, NGS is capable of detecting a range of changes from single nucleotide variants to short insertions/deletions in all regions of the genome. NGS and ddPCR are quantitative methods, whereas PNA–ddPCR, though more sensitive is only qualitative. Among the original 15 SWS patients^7^, the *GNAQ* mutation (c.548G > A) in brain lesions was found in 14 patients (93%) using ddPCR and PNA–ddPCR. The mutation detection rate was therefore elevated from 80% (12/15) to 93% (14/15) by the use of ddPCR–based technologies.

We found the *GNAQ* mutation (c.548G > A) in brain lesions, blood leukocytes and saliva. Brain vascular system and lymphocytes in saliva and blood leukocytes are all of the mesodermal origin^14^. In embryogenesis, the mesoderm subsequently differentiates into blood islands and haemangioblasts in yolk sacs. In turn, haemangioblasts differentiate into endothelial cells and haemogenic endothelium^14^,^15^,^16^. Adult haematopoietic stem cells are differentiated from a particular type of haemogenic endothelium in aorta–gonad–mesonephros (AGM)^15^,^16^,^17^,^18^. These haematopoietic stem cells move to bone marrow after birth and continue haematopoiesis throughout life. Vascular malformations in SWS patients originate from endothelial cells. The *GNAQ* somatic mutation (c.548G > A) may occur in a haemangioblast or early endothelial cell. It is unknown whether the presence of a *GNAQ* mutation in blood or saliva is pathologically important. However, it is possible that cells harboring the mutation in blood leukocytes or saliva lymphocytes actively disseminate SWS lesions.

An extremely low–prevalence *GNAQ* mutation was found in two brain lesions and two blood samples in our 15 patients using PNA–ddPCR. Such low frequencies of mutant allele could be explained by an unusually low level of lesion infiltration contained in tissue sections used for DNA extraction. It is particularly important that we were able to find a *GNAQ* mutation in blood and saliva samples. A highly sensitive method of detecting the mutation in DNA of easily accessible tissues may be useful for developing a diagnostic method for SWS. The combination of two existing technologies described in this study could also be used for treatment monitoring or identification of relapse in some melanomas with the same *GNAQ* mutation^19^,^20^.

## Methods

### Patient and sample selection

This study was approved by the Institutional Review Board of Yokohama City University School of Medicine and Juntendo University Graduate School of Medicine. All experiments were carried out in accordance with the institutional guidelines. Written informed consent was obtained from patients or parents of pediatric patients. A total of 15 patients clinically diagnosed with SWS, who were previously analysed by NGS only^7^, were examined again in our study. Paired sets of peripheral blood leukocytes/saliva and surgically resected brain tissues were obtained from the 15 SWS patients^7^. In addition to the patients previously screened by NGS, we also included an additional cohort of 25 patients clinically diagnosed with SWS for whom only blood leukocytes were available and 30 normal controls in whom only blood leukocytes were available.

### DNA extraction

Genomic DNA from RNAlater (Thermo Fisher Scientific, Waltham, MA, USA)–treated brain tissue blocks, peripheral blood leukocytes and saliva was extracted using Puregene Core Kit A (Qiagen, Valencia, CA, USA), PAXgene Blood DNA kit (Qiagen) and Oragene (DNA Genotek Inc., Ottawa, ON, Canada), respectively, according to the manufacturer’s instruction. DNA concentration was measured using the Qubit dsDNA BR assay kit (Thermo Fisher Scientific) according to the manufacturer’s instruction.

### Control DNAs

The positive and negative control DNAs were necessary to set Tm values and minimise background for the ddPCR assay. A 178–bp fragment harboring c.548G > A in *GNAQ* or a corresponding wild–type allele was amplified using primers as shown in Table 3 and brain section DNA (as a template) from a SWS patient. The fragment was cloned in TOPO pCR2.1 Vector using a TOPO TA Cloning Kit (Life Technologies, Carlsbad, CA, USA). The presence of the inserted fragment in each clone was confirmed by Sanger sequencing.

### Confirmation of detection limit in ddPCR analysis

ddPCR was performed using a Droplet Digital PCR XQ200 system (Bio–Rad Laboratories, Hercules, CA, USA). The region–specific primers and customised locked nucleic acid (LNA) probes for wild–type and mutant alleles were purchased from Integrated DNA Technology (Coralville, IA, USA). Sequences of primers and probes were described in Table 3. We first tested ddPCR using serial dilutions of DNA of wild–type and mutant plasmids (each 4104 bp in size). One nanogram of plasmid DNA should contain 2.22 × 10^8^ copies (one plasmid clone = 4.50 × 10^−9^ ng). Mutant and wild–type clones were mixed serially in different ratios: 10, 5, 1, 0.5, 0.25 and 0.1% (mutant/mutant + wild–type). Mixed plasmid DNA (1.5 × 10^−5^ ng equivalent to 3.0 × 10^3^ copies) was added to a 20–μL PCR mixture containing 10 μL 2x ddPCR Supermix for probes (No dUTP; Bio–Rad), 900 nM target–specific PCR primers, 250 nM mutant–specific (FAM) and wild–type–specific (HEX) LNA probes. Twenty microliters of PCR mixture and 70 μL Droplet generation oil for Probes (Bio–Rad) were mixed and droplet generation was carried out using a QX100 Droplet Generator (Bio–Rad) according to the manufacturer’s manual. The droplet emulsion was thermally cycled in the following conditions: denaturing at 95 °C for 10 min, 40 cycles of PCR at 94 °C for 30 s and 57 °C for 2 min, and a final extension at 98 °C for 10 min. PCR amplification in droplets was confirmed using QX200 Droplet Reader (Bio–Rad). Threshold was determined by comparing wild–type and no–template ddPCR results. All data were evaluated above the threshold. All experiments were performed in triplicate.

### Evaluation of a *GNAQ* mutation by ddPCR

The somatic *GNAQ* mutation (c.548G > A), which was previously evaluated using NGS in 15 patients with SWS^7^, was re–evaluated by ddPCR. Approximately 20 ng of genomic DNA (which is equivalent to 6.0 × 10^3^ copies of a haploid genome) from paired brain and either blood leukocytes or saliva samples from SWS patients was similarly evaluated by ddPCR (Table 2 and Supplementary Table S2).

### Qualitative evaluation using PNA–ddPCR

Wild–type–specific PNA corresponding to *GNAQ* c.548G was purchased from Fasmac (Atsugi, Kanagawa, Japan). Primer and probe sequences are described in Table 3.

To evaluate the sensitivity of PNA–ddPCR, the detection limit was determined in a similar way to ddPCR. Mutant and wild–type clones were mixed serially in different ratios: 1, 0.5, 0.25, 0.1, 0.05 and 0.025% (mutant/mutant + wild–type; Fig. 1a). Mixed plasmid DNA (1.5 × 10^−5^ ng, 3.0 × 10^3^ copies) was used as a template in the first PCR mixture. The PCR mixture was prepared in a 10–μL volume containing 1 μL of 10x PCR Buffer (Takara, Otsu, Shiga, Japan), 0.8 μL of 2 mM dNTP Mixture (Takara), 0.05 μL of Takara Ex Taq HS (Takara), 1 μM forward and reverse primers, and 5 μM PNA together with mixed plasmid DNA. PCR conditions were as follows: denaturing at 94 °C for 2 min; 15 cycles at 94 °C for 30 s and 60 °C for 10 s; and extension at 72 °C for 3 min. Then, 1.5 μL PCR product was added to the ddPCR solution containing 10 μL 2x ddPCR Supermix for probes (No dUTP; Bio–Rad), 900 nM target–specific PCR primers and 250 nM mutant–specific (FAM) LNA probes. ddPCR was performed as described above. We also performed PNA–ddPCR using DNA from 30 healthy controls to confirm whether the threshold was appropriate.

### The confirmation of detection limit of PNA–ddPCR assay

The *GNAQ* c.548G > A mutation was confirmed by PNA–ddPCR using genomic DNA of peripheral blood leukocytes from 40 SWS patients. Ten nanograms of genomic DNA (equivalent to 3.0 × 10^3^ copies of a haploid genome) was added to the PCR mixture. PCR conditions were as follows: denaturing at 94 °C for 2 min; 20 cycles at 94 °C for 30 s and 60 °C for 10 s; and extension at 72 °C for 3 min. Each 1.5 μL of PCR product was evaluated by PNA–ddPCR.

### Data analysis

The data was analysed using QuantaSoft (version 1.7.4.0917, Bio–Rad). Absolute quantification mode was used for all ddPCR measurements. Absolute quantification was based on the following underlined formula:


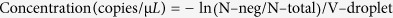


[N–neg = the number of total negative droplets in a well; N–total = the number of total droplets in a well; V–droplet = the volume of a droplet (0.85 nl, fixed)].

The threshold for positive amplification was determined based on the results of no template control, mutant and wild–type cloning plasmids and control genomic DNA. Fractional abundance (FA) was calculated as follows:


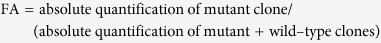


As for PNA–ddPCR, the threshold of positive amplification was set to more than 1,000 mutant droplet showing a mutant–specific signal amplitude > 7,700.

## Additional Information

**How to cite this article**: Uchiyama, Y. *et al*. Ultra-sensitive droplet digital PCR for detecting a low-prevalence somatic *GNAQ* mutation in Sturge-Weber syndrome. *Sci. Rep.* **6**, 22985; doi: 10.1038/srep22985 (2016).

## Supplementary Material

Supplementary Information

## Figures and Tables

**Table 1:** Mutant clone ratios detected by ddPCR.

Mut clone ratios	Mut copy number*	Mut drop number	Wt drop number	ACD	FA (%)	FA range (%)
10%	144	101	707	16444	12.3	14.5–10.0
10%	166	114	754	16252	12.9	15.1–10.7
10%	150	103	1046	16196	8.7	10.3–7.1
5%	74	50	771	15951	6	7.6–4.3
5%	74	49	714	15547	6.3	8.0–4.6
5%	66	48	1050	16941	4.2	5.4–3.1
1%	14.4	9	681	14684	1.3	2.1–0.4
1%	10.2	7	729	16037	0.9	1.6–0.2
1%	16	11	1182	16282	0.9	1.4–0.4
0.50%	8.6	5	576	13723	0.8	1.6–0.1
0.50%	4.6	3	552	15250	0.5	1.2–0
0.50%	8.8	6	1031	16030	0.56	1.02–0.1
0.25%	5	3	679	14038	0.43	0.95–0
0.25%	3.6	2	631	12837	0.31	0.78–0
0.25%	4.2	3	1104	16872	0.26	0.58–0
0.10%	0	0	772	16230	0	0–0
0.10%	1.4	1	788	16400	0.12	0.42–0
0.10%	3.4	2	975	13931	0.2	0.50–0

^*^: Calculated copy number by Quantasoft^™^, Mut: mutant, Wt: wild–type, drop: droplet, ACD: accepted droplets, FA: fractional abundance, PFA: Poisson fractional abundance.

**Table 2:** ddPCR and PNA–ddPCR results of 15 paired SWS patients.

Sample	Sample type	Mut copy number	Mut drop	Wt drop	ACD	FA (%)	deep–seq	PNA-ddPCR
SWS1	brain	246.67 ( 9.29 )	138.33 ( 15.11 )	2188.00 ( 155.86 )	13208.33 ( 1009.30 )	**5.50** ( 0.24 )	6.0	+
	blood	7.80 ( 4.82 )	4.33 ( 2.87 )	2520.67 ( 88.51 )	12677.67 ( 740.68 )	**0.15** ( 0.10 )	0.03	+new
SWS2	brain	80.67 ( 15.86 )	52.00 ( 8.60 )	1097.67 ( 13.47 )	15316.00 ( 839.64 )	**4.37** ( 0.66 )	4.0	+
	blood	8.87 ( 5.01 )	5.33 ( 3.09 )	4008.33 ( 2093.11 )	13836.00 ( 611.36 )	**0.11** ( 0.06 )	0.05	+new
SWS3	brain	9.60 ( 3.37 )	4.67 ( 1.25 )	2876.67 ( 125.27 )	11946.33 ( 1546.93 )	**0.14** ( 0.04 )	0.03	+new
	blood	1.53 ( 1.23 )	1.00 ( 0.82 )	3066.33 ( 166.55 )	14435.33 ( 873.95 )	**0.04** ( 0.02 )	0.03	−
SWS4	brain	11.33 ( 5.56 )	6.67 ( 3.86 )	2894.67 ( 128.23 )	13326.00 ( 1169.36 )	**0.20** ( 0.11 )	0.16	+new
	blood	2.87 ( 2.29 )	2.00 ( 1.63 )	2356.33 ( 450.70 )	14885.67 ( 1623.49 )	**0.11** ( 0.02 )	0.03	−
SWS5	brain	222.67 ( 17.00 )	148.00 ( 15.12 )	2979.67 ( 215.29 )	15683.33 ( 423.06 )	**4.33** ( 0.53 )	5.0	NP
	blood	4.73 ( 2.46 )	3.00 ( 1.63 )	5104.00 ( 1862.52 )	14706.33 ( 645.52 )	**0.05** ( 0.04 )	0.04	−
SWS6	brain	331.33 ( 10.50 )	207.67 ( 4.03 )	2772.33 ( 121.38 )	14858.67 ( 354.37 )	**6.40** ( 0.22 )	6.0	NP
	blood	3.60 ( 1.88 )	2.33 ( 1.25 )	3423.67 ( 513.43 )	15444.00 ( 1326.28 )	**0.06** ( 0.03 )	0.04	−
SWS7	brain	177.33 ( 16.76 )	111.33 ( 13.89 )	2674.33 ( 173.12 )	14798.67 ( 634.96 )	**3.63** ( 0.33 )	4.0	NP
	blood	1.60 ( 1.40 )	1.00 ( 0.82 )	4894.00 ( 2358.55 )	15173.67 ( 1153.39 )	**0.03** ( 0.02 )	0.04	−
SWS8	brain	295.33 ( 8.22 )	181.00 ( 7.12 )	2616.33 ( 124.93 )	14501.33 ( 320.86 )	**5.97** ( 0.12 )	6.0	+
	saliva	51.33 ( 5.25 )	35.33 ( 4.78 )	4856.33 ( 533.45 )	16400.33 ( 1350.59 )	**0.61** ( 0.06 )	0.44	+
SWS9	brain	346.00 ( 17.05 )	178.00 ( 7.26 )	2339.00 ( 69.37 )	12196.67 ( 828.45 )	**6.47** ( 0.09 )	8.0	NP
	blood	1.07 ( 0.75 )	0.67 ( 0.47 )	3111.00 ( 107.34 )	14228.33 ( 241.84 )	**0.02** ( 0.01 )	0.03	−
SWS10	brain	420.00 ( 41.09 )	232.00 ( 19.44 )	2565.00 ( 166.28 )	13152.67 ( 250.69 )	**7.60** ( 0.08 )	8.0	NP
	blood	3.40 ( 2.70 )	1.67 ( 1.25 )	2381.67 ( 237.10 )	12761.67 ( 1533.49 )	**0.07** ( 0.05 )	0.04	−
SWS11	brain	4.60 ( 3.15 )	2.67 ( 1.70 )	2894.00 ( 226.81 )	14169.33 ( 1065.11 )	**0.09** ( 0.06 )	0.03	−
	blood	0.00 ( 0 )	0.00 ( 0 )	1078.33 ( 379.28 )	13129.00 ( 915.10 )	**0.00** ( 0 )	0.03	−
SWS12	brain	136.67 ( 12.26 )	73.00 ( 20.22 )	2320.67 ( 629.80 )	12492.67 ( 2597.01 )	**2.80** ( 0.08 )	4.0	NP
	blood	0.87 ( 1.23 )	0.33 ( 0.47 )	1372.33 ( 544.09 )	10545.00 ( 2524.01 )	**0.03** ( 0.04 )	0.04	−
SWS13	brain	170.67 ( 29.23 )	87.67 ( 10.62 )	2099.00 ( 302.03 )	12690.00 ( 3164.55 )	**3.70** ( 0.08 )	4.0	NP
	blood	3.67 ( 3.30 )	2.33 ( 2.05 )	2414.67 ( 78.47 )	14800.00 ( 921.06 )	**0.09** ( 0.08 )	0.03	−
SWS14	brain	66.67 ( 4.11 )	43.00 ( 3.56 )	481.67 ( 24.64 )	15137.67 ( 854.48 )	**8.07** ( 0.29 )	9.0	NP
	blood	1.60 ( 1.23 )	1.00 ( 0.82 )	2086.67 ( 108.02 )	14751.33 ( 1099.24 )	**0.04** ( 0.03 )	0.04	−
SWS15	brain	94.00 ( 21.42 )	55.33 ( 4.64 )	1485.67 ( 112.35 )	14378.33 ( 2276.07 )	**3.43** ( 0.45 )	4.0	NP
	blood	3.27 ( 1.23 )	2.00 ( 0.82 )	2560.33 ( 131.76 )	14101.00 ( 846.50 )	**0.07** ( 0.02 )	0.03	−

Mut: mutant, Wt: wild–type, ACD: number of accepted droplets, FA: fractional abundance, PFA: Poisson fractional abundance, deep–seq: mutant ratio by deep sequencing (previously reported)^7^, NP: not performed, + : positive, −: negative, + new: positive only by PNA–ddPCR (not by the other methods). ddPCR was performed in triplicate. SD values are shown in parentheses after the mean values. PNA–ddPCR was performed in triplicate where “ +new” is shown.

**Table 3:** Sequences of primers and probes.

Primer information	Primer and probe sequences
PCR primers amplifying a 178-bp fragment containing a *GNAQ* mutation which was cloned into TA vector.	5′-ATTGTGTCTTCCCTCC−3′ (forward) 5′-GGTTTCATGGACTCAG−3′ (reverse)
ddPCR primers amplfying a 114-bp fragment containing a *GNAQ* mutation	5′-CCTGCCTACGCAACAAGAT−3′ (forward) 5′-AGGTTTCATGGACTCAGTTACTAC-3′ (reverse)
LNA probes	5′(HEX)−TGGGGAC + T + C + GAAC-3′(IABkFQ) (wild−type)
	5′(HEX)−TGGGG + AC + T + T + GAAC-3′(IABkFQ) (mutant)
PNA probe (wild type)	5′-GGGACTCGAACTCTA-3′
