# Corrigendum: Ultra–sensitive droplet digital PCR for detecting a low–prevalence somatic GNAQ mutation in Sturge–Weber syndrome

**DOI:** 10.1038/srep39897

**Published:** 2017-01-12

**Authors:** Yuri Uchiyama, Mitsuko Nakashima, Satoshi Watanabe, Masakazu Miyajima, Masataka Taguri, Satoko Miyatake, Noriko Miyake, Hirotomo Saitsu, Hiroyuki Mishima, Akira Kinoshita, Hajime Arai, Ko–ichiro Yoshiura, Naomichi Matsumoto

Scientific Reports 6: Article number: 2298510.1038/srep22985; published online: 03 09 2016; updated: 01 12 2017

This Article contains a typographical error in the name of fluorescence dye for the mutant LNA probe in Table 3. The correct Table 3 appears below.

## Figures and Tables

**Table 3:** 

Primer information	Primer and probe sequences
PCR primers amplifying a 178-bp fragment containing a GNAQ mutation which was cloned into TA vector.	5′−ATTGTGTCTTCCCTCC−3′ (forward)
	5′−GGTTTCATGGACTCAG−3′ (reverse)
ddPCR primers amplfying a 114-bp fragment containing a GNAQ mutation	5′−CCTGCCTACGCAACAAGAT−3′ (forward)
	5′−AGGTTTCATGGACTCAGTTACTAC−3′ (reverse)
LNA probes	5′(HEX)−TGGGGAC+T+C+GAAC−3′(IABkFQ) (wild−type)
	5′(FAM)−TGGGG+AC+T+T+GAAC−3′(IABkFQ) (mutant)
PNA probe (wild type)	5′−GGGACTCGAACTCTA−3'

