# Supplementary material for: Ultra–sensitive droplet digital PCR for detecting a low–prevalence somatic GNAQ mutation in Sturge–Weber syndrome
Source: Sci Rep. 2016 Mar 9;6:22985. doi: 10.1038/srep22985 (PMC4783707; doi:10.1038/srep22985)
Supplement: Supplementary Information [file srep22985-s1.pdf]

Supplementary Information

Ultra–sensitive droplet digital PCR for detecting a low–prevalence somatic *GNAQ* mutation in Sturge–Weber syndrome

Yuri Uchiyama[1,2], Mitsuko Nakashima[1], Satoshi Watanabe[3], Masakazu Miyajima[4], Masataka Taguri[5], Satoko Miyatake[1], Noriko Miyake[1], Hirotomo Saitsu[1], Hiroyuki Mishima[3], Akira Kinoshita[3], Hajime Arai[4], Ko–ichiro Yoshiura[3], and Naomichi Matsumoto[1*]

[1]Department of Human Genetics, Yokohama City University Graduate School of Medicine, Yokohama, Japan; [2]Department of Medical and Clinical Science, Gunma University Graduate School of Medicine, Gunma, Japan; [3]Department of Human Genetics, Nagasaki University Graduate School of Biomedical Sciences, Sakamoto, Nagasaki, Japan; [4]Department of Neurosurgery, Juntendo University Graduate School of Medicine, Tokyo, Japan, [5]Department of Biostatistics, Graduate School of Medicine, Yokohama City University, Yokohama, Japan

Table of Contents

Supplementary Data
Table S1. Probability of the zero Mutant droplet number according to different values of (*p*, *N*, *R*) (%)
Table S2. ddPCR and PNA–ddPCR results of 25 SWS patients.

Supplementary Data

A statistical consideration of the detection limit of ddPCR

We assume that the proportion of the mutant allele frequencies (fractional abundance; FA) can be approximately calculated using the following formula:

$$FA = \text{Mutant droplet number} / N,$$

where N =(Mutant droplet number + Wild-type droplet number). Let us denote p as the true proportion of Mutant. Then, Mutant droplet number will be distributed according to the binomial distribution with parameters N and p. With the replication number of the experiment R, the probability of the zero Mutant droplet number can be calculated as follows:

$$q = (1 - p)^{NR}$$

Using the above formula, we calculated q according to different values of (p, N, R). The result is summarized in the following Table S1.

From the table, the worst probability of the zero Mutant droplet number with p = 0.25% is 0.7% (N=2000, R = 1) while that with p = 0.10% is 13.5%. Thus, it will be reasonable to set the detection limit at 0.25%.

Table S1. Probability of the zero Mutant droplet number according to different values of (*p*, *N*, *R*) (%).

p	*R*	*N* 500	1000	2000	4000	6000
5%	1	0.0	0.0	0.0	0.0	0.0
	2	0.0	0.0	0.0	0.0	0.0
	3	0.0	0.0	0.0	0.0	0.0
1%	1	0.7	0.0	0.0	0.0	0.0
	2	0.0	0.0	0.0	0.0	0.0
	3	0.0	0.0	0.0	0.0	0.0
0.50%	1	8.2	0.7	0.0	0.0	0.0
	2	0.7	0.0	0.0	0.0	0.0
	3	0.1	0.0	0.0	0.0	0.0
0.25%	1	28.6	8.2	0.7	0.0	0.0
	2	8.2	0.7	0.0	0.0	0.0
	3	2.3	0.1	0.0	0.0	0.0
0.10%	1	60.6	36.8	13.5	1.8	0.2
	2	36.8	13.5	1.8	0.0	0.0
	3	22.3	5.0	0.2	0.0	0.0

Table S2. ddPCR and PNA–ddPCR results of 25 SWS patients.

Sample	sample type	Mut copy number	Mut drop number	Wt drop number	ACD	FA (%)	PFA range (%)	PNA-ddPCR
SWS16	blood	2.4	1	2250	10221	0.04	0 - 0.13	-
SWS17	blood	4.4	2	2724	10926	0.06	0 - 0.16	-
SWS18	blood	0	0	2895	12675	0	0	-
SWS19	blood	3.6	2	2626	12947	0.07	0 - 0.17	-
SWS20	blood	1.8	1	2978	12712	0.029	0 - 0.10	-
SWS21	blood	1.8	1	3484	13727	0.025	0 - 0.08	-
SWS22	blood	2.2	1	2488	10960	0.04	0 - 0.12	-
SWS23	blood	5.8	3	2858	12269	0.09	0 - 0.20	-
SWS24	blood	1.8	1	3216	13179	0.027	0 - 0.09	-
SWS25	blood	4.2	2	2757	11429	0.06	0 - 0.16	-
SWS26	blood	1.8	1	3023	13023	0.029	0 - 0.10	-
SWS27	blood	3.4	2	3413	14055	0.05	0 - 0.13	-
SWS28	blood	0	0	2656	12822	0	0	-
SWS29	blood	6.6	4	3090	14092	**0.11**	0 - 0.23	+ new
SWS30	blood	0	0	2132	15019	0	0	-
SWS31	blood	0	0	3187	14285	0	0	-
SWS32	blood	1.8	1	2843	13436	0.03	0 - 0.11	-
SWS33	blood	0	0	3068	15080	0	0	-
SWS34	blood	2.4	1	2049	10099	0.04	0 - 0.15	-
SWS35	blood	1.6	1	1860	14146	0.05	0 - 0.17	-
SWS36	blood	1.6	1	3326	14722	0.027	0 - 0.09	-
SWS37	blood	3	2	2928	15207	0.06	0 - 0.16	-
SWS38	blood	4.8	3	3108	14661	0.09	0 - 0.19	-
SWS39	blood	0	0	3113	14782	0	0	-
SWS40	blood	1.6	1	2439	13880	0.04	0 - 0.13	-

Mut: Mutant, Wt: Wild-type, ACD: number of accepted droplets, FA: Fractional abundance, PFA: Poisson fractional abundance, NP: Not performed, +: positive, -: negative, + new: positive only by PNA-ddPCR.
